# Cancer as a channelopathy: ion channels and pumps in tumor development and progression

**DOI:** 10.3389/fncel.2015.00086

**Published:** 2015-03-17

**Authors:** Alisa Litan, Sigrid A. Langhans

**Affiliations:** Nemours Center for Childhood Cancer Research, Alfred I. duPont Hospital for ChildrenWilmington, DE, USA

**Keywords:** sodium channel, potassium channel, Na-K-ATPase, migration, actin, signaling

## Abstract

Increasing evidence suggests that ion channels and pumps not only regulate membrane potential, ion homeostasis, and electric signaling in excitable cells but also play important roles in cell proliferation, migration, apoptosis and differentiation. Consistent with a role in cell signaling, channel proteins and ion pumps can form macromolecular complexes with growth factors, and cell adhesion and other signaling molecules. And while cancer is still not being cataloged as a channelopathy, as the non-traditional roles of ion pumps and channels are being recognized, it is increasingly being suggested that ion channels and ion pumps contribute to cancer progression. Cancer cell migration requires the regulation of adhesion complexes between migrating cells and surrounding extracellular matrix (ECM) proteins. Cell movement along solid surfaces requires a sequence of cell protrusions and retractions that mainly depend on regulation of the actin cytoskeleton along with contribution of microtubules and molecular motor proteins such as mysoin. This process is triggered and modulated by a combination of environmental signals, which are sensed and integrated by membrane receptors, including integrins and cadherins. Membrane receptors transduce these signals into downstream signaling pathways, often involving the Rho GTPase protein family. These pathways regulate the cytoskeletal rearrangements necessary for proper timing of adhesion, contraction and detachment of cells in order to find their way through extracellular spaces. Migration and adhesion involve continuous modulation of cell motility, shape and volume, in which ion channels and pumps play major roles. Research on cancer cells suggests that certain ion channels may be involved in aberrant tumor growth and channel inhibitors often lead to growth arrest. This review will describe recent research into the role of ion pumps and ion channels in cell migration and adhesion, and how they may contribute to tumor development.

## Introduction

**Figure 1:**
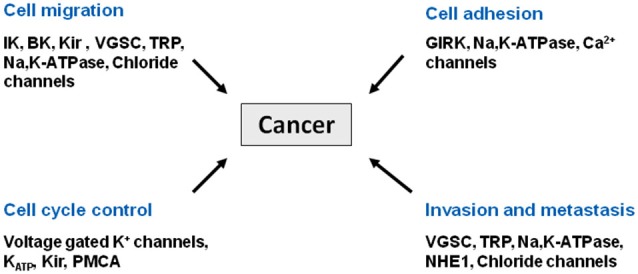
**Cancer as a channelopathy**. Mutations, loss of expression/function and aberrant expression/function of ion channels and transporters have been linked to various cancers. Aberrant cell migration, cell adhesion, and cell cycle control all contribute to the invasive phenotype associated with metastasis.

Cancer is a leading cause of death worldwide. According to the American Cancer Society, more than 1.6 million new cases were diagnosed in 2013, and one in four deaths in the US is cancer related. Most cancers are treated with surgery, chemotherapy, and/or radiation therapy. Although these clinical measures have proven their efficacy in many cases, patients often experience debilitating side effects that significantly reduce their quality of life. Moreover, cancer relapse with treatment resistance underscores the urgent need to identify novel molecular targets for the development of alternative therapies. Recently, the role of ion channels in driving malignant cancer cell behavior has been revealed and much has been learned from brain tumors (Figure 1). Changes in channel expression have been observed primarily in glioblastoma, the most aggressive malignant brain tumor arising from astrocytes (Sontheimer, 2008) and have also been seen in medulloblastoma, the most common pediatric brain tumor that originates in the cerebellum (Hatten and Roussel, 2011). With our increasing knowledge of ion channels and pumps in tumor development cancer is being classified as a channelopathy, or a disease brought about by disturbed function of ion channels, often due to dysregulation of channel expression (transcriptional channelopathy) or other modifications resulting in altered function (Pedersen and Stock, 2013; Djamgoz et al., 2014) (and articles therein).

The proteins that transport ions across cell membranes fall into two general classes: ion channels, through which ions move down their concentration and electrical gradients, and ion pumps that use energy to actively move ions against those gradients (Gadsby, 2009). About 13% of currently known drugs whose primary therapeutic targets are ion channels are being used for the treatment of a variety of human conditions, including cardiovascular and neurological disorders (Overington et al., 2006). Some examples include lidocaine, a local anesthetic that targets sodium channels, verapamil, a drug used to treat hypertension and targets calcium channels, riluzole which is used to treat amyotrophic lateral sclerosis and targets sodium channels, and the class of drugs that target sodium channels in the brain to treat epilepsy such as carbamazepine and topiramate (Camerino et al., 2007). With our evolving understanding of the molecular mechanisms of channelopathies, ion channels have now become a promising player for the development of novel anticancer therapies. In this review we will discuss a variety of classes of channels and pumps and their relationship to cancer development and progression including sodium, potassium, chloride, and calcium channels, and ion transport pumps.

## Overview of Ion Channels and Pumps with Dysregulated Expression in Cancer Cells

### Potassium Channels

Potassium channels can be divided into four classes based on their domain structure and activation mechanisms. Voltage-gated potassium channels (Kv) are the largest subset of potassium channels, and they are gated by changes in membrane potential. Calcium-activated potassium channels (KCa) are activated by intracellular calcium. According to their conductance, KCa channels can be further divided into big conductance (BK), intermediate conductance (IK), and small conductance (SK) channels. KV and KCa channels share a similar structure—they are composed of four pore-lining α-subunits, each with six transmembrane domains and one pore-forming region, except for the BK channel, which has one additional transmembrane segment in the N terminus. Transmembrane regions 1–4 represent the voltage-sensing domains for Kv channels (Huang and Jan, 2014). Inward rectifying potassium channels (Kir) possess two transmembrane segments flanking one pore loop in each of the four α-subunits. Two-pore domain potassium channels (K2P) have two pore domains per α-subunit. Two α-subunits form a K2P channel that is usually constitutively open as a “leak channel” for maintaining a negative membrane potential (Huang and Jan, 2014). Multiple studies have reported dysregulated potassium channel expression in human cancer. Overexpression of K_V_1.1 is seen in medulloblastoma (Northcott et al., 2012), elevated K_V_1.3 can be detected in multiple malignancies including breast, colon, and prostate cancer (Comes et al., 2013), and overexpression of a specific splice variant of the BK channel correlates with the malignancy grade of glioma (Liu et al., 2002). Altered expression of the intermediate-conductance calcium activated channel KCa3.1 has also been shown in glioblastoma (Catacuzzeno et al., 2012). K_V_1.5 channels are dysregulated in multiple cancers including lymphomas, astrocytomas, oligodendrogliomas, and glioblastomas (Comes et al., 2014). Expression levels of K_V_10.1 (EAG1, voltage gated eag related subfamily H, member 1) are lower in glioblastoma multiforme and in malignant brain tumors than in normal brain tissue, and expression of the EAG1 channel is inversely related to malignancy of the tumor (Patt et al., 2004). Human ether-a-go-go related gene (hERG) K^+^ channel, also known as K_V_11.1, is constitutively expressed in neuroblastoma, and a molecular complex between β_1_-integrins and hERG channels regulates adhesion-dependent differentiation of neuroblastoma cells (Cherubini et al., 2005). And expression of Kir4.1 channels in glioma cells impaired cell growth (Higashimori and Sontheimer, 2007).

### Sodium Channels and Exchangers

Voltage gated sodium channels (VGSCs) are responsible for the rising phase of the action potential in the majority of electrically excitable cells and are therefore important in impulse generation and propagation. VGSCs comprise a multi-gene family of at least nine different functional members (Na_V_1.1–1.9) coding for the pore-forming α-subunits. There are also four auxiliary β-subunits, of which one or two at a time can associate with an α-subunit and modulate channel expression and activity in the plasma membrane. Recently, VGSCs have been found to have relatively high expression levels in a range of cell types that are considered non excitable, including immune cells, fibroblasts and cancer cells (Brackenbury, 2012). Moreover, several individual Na_V_ isoforms are differentially expressed in different human cancers. These include Na_V_1.5 in astrocytoma, breast and colon cancers (Chioni et al., 2010; Brisson et al., 2013; Driffort et al., 2014; Xing et al., 2014), Na_V_1.6 in cervical cancer, and Na_V_1.7 in breast, prostate and non-small cell lung cancers (Fraser et al., 2014). Another group of sodium channels is the epithelial sodium channel family, located in the apical membrane of polarized epithelial cells. High grade gliomas express multiple members of the epithelial sodium channel family, and there is an elevated expression of the acid-sensing ion channel 1 (ASIC1) in glioblastoma cells compared to primary astrocytes (Kapoor et al., 2009).

In addition to these sodium channel families, there is also a group of exchanger proteins that involve transport of sodium ions. Some of these exchanger proteins include the Na^+^/H^+^ exchanger (NHE1), the Na^+^, ${\text{HCO}}_{\text{3}}^{\text{−}}$ contransporter, and the Na^+^, K^+^, 2Cl^−^ cotransporter (NKCC). All of these proteins utilize the Na^+^ electrochemical gradient to drive the transport of other ions, and they are all important in maintaining cellular pH. The NHE1 is a ubiquitously expressed transporter that contributes to cell volume regulation and cytoplasmic pH homeostasis. In cancer NHE1 is upregulated and/or overexpressed in various tumors and plays a fundamental role in malignant invasion by altering the metabolic environment and cell invasiveness (Cardone et al., 2005; Stock et al., 2012). For example, NHE is involved in breast cancer and melanoma invasion and metastasis (Stüwe et al., 2007; Amith and Fliegel, 2013; Vahle et al., 2014) indicating that NHE1 may be a suitable target for anticancer therapy (Stock et al., 2012). Aside from NHE1, additional exchangers have been shown to be affected in cancer. For example, the Na^+^/${\text{HCO}}_{\text{3}}^{\text{−}}$ contransporter is upregulated by human epidermal growth factor receptor 2 (HER2) signaling in breast cancer cells (Gorbatenko et al., 2014), and NKCC modulates glioma cell migration through regulation of focal adhesions and cell volume (Garzon-Muvdi et al., 2012).

### Chloride Channels

The CLC and the cystic fibrosis transmembrane conductance regulator (CFTR) channels are distinct classes of chloride channels. Functions of chloride channels range from ion homeostasis to cell volume regulation and regulation of excitable cells. These channels have 10–12 transmembrane domains, and are found both in the plasma membrane and the membranes of various organelles. Dysregulation of chloride channels has been reported in multiple cancer types. Expression of CLCA1 and CLCA2 is downregulated in human colorectal cancer, revealing a possible tumor suppressor role for these proteins (Bustin et al., 2001). Changes in glioma-specific chloride current are linked to the cell cytoskeleton, and cytoskeletal rearrangements associated with cell division lead to change in chloride channel activity (Ullrich and Sontheimer, 1997), and regulate glioblastoma cell invasiveness (Turner and Sontheimer, 2014).

### Calcium Channels

Ca^2+^ is a ubiquitous second messenger, and is an important signaling molecule for several fundamental cell processes including cell cycle control, migration, and apoptosis. Some human diseases that have been associated with Ca^2+^ homeostasis include cancer, Alzheimer’s disease, and cardiovascular disease (Chen et al., 2013). Regulation of intracellular Ca^2+^ involves both Ca^2+^ entry from the extracellular space and Ca^2+^ release from intracellular stores in the endoplasmic reticulum (ER) or mitochondria. Calcium channels not only allow facilitated diffusion of Ca^2+^ down its concentration gradient, they also provide for the flow of Ca^2+^ out of the ER into the cell cytosol through ER Ca^2+^ channels. Plasma membrane channels involved in the influx of Ca^2+^ into the cell include voltage-gated Ca^2+^ and transient receptor potential (TRP) ion channels. There are multiple types of calcium channels that are differentially regulated in various cancer types. TRPC3 is elevated in some breast and ovarian tumors, and its silencing reduces ovarian cancer cell line proliferation *in vitro* and tumor formation *in vivo* (Aydar et al., 2009). TRPC6 expression is elevated in breast, liver, and stomach cancers and in glioma. Silencing of TRPC6 reduces proliferation of some esophageal and breast cancer cell lines and glioma cells (Ding et al., 2010). The expression level of TRPM7 and formation of metastases are positively correlated in breast cancer, suggesting that TRPM7 contributes to a migratory and invasive phenotype. T-type voltage gated Ca^2+^ channels are expressed in normal tissues as well as in various cancers such as breast carcinoma, retinoblastoma, neuroblastoma, and glioma (Zhang et al., 2014). Increased expression of the α1 subunit of T-type channels contributes to abnormal proliferation of glioblastoma cells, siRNA-mediated knockdown of the α1 subunit decreases proliferation of these cells, and pharmacological blockade of T-type channels decreases tumor growth (Zhang et al., 2014).

### Ion Exchangers

The P-type ATPases are a major class of ion pumps and are characterized by an aspartate residue in the cytoplasmic domain that gets phosphorylated by ATP once transported ions have entered the binding pocket (Gadsby, 2009). Some examples of P-type ATPases are the Sodium potassium ATPase (Na,K-ATPase), the sarcoplasmic and endoplasmic reticulum Ca-ATPase (SERCA), the H,K-ATPase and the H-ATPase. To date, the best characterized pumps that have been linked to cancer are SERCA and the Na,K-ATPase. SERCA ATPases are important regulators of intracellular calcium concentration. They enable muscle contraction and relaxation, and control calcium signaling pathways (Yatime et al., 2009). SERCA also has recently been identified as a therapeutic target in certain types of cancer. SERCA inhibition induces a G0/G1 arrest in NOTCH1-mutated human leukemia cells, introducing SERCA as a potential therapeutic target in cancers associated with NOTCH1 mutations (Roti et al., 2013). SERCA inhibition through a thapsigargin prodrug is being tested as a potential treatment for prostate cancer by inducing apoptosis (Denmeade et al., 2003). The Na,K-ATPase is a ubiquitously expressed integral membrane protein that carries out the extrusion and uptake of Na^+^ and K^+^ ions across the plasma membranes of cells of most higher eukaryotes. It is composed of a catalytic α-subunit with ten transmembrane segments and a heavily glycosylated β-subunit. The pump is critical in maintaining a physiological electrochemical gradient that is essential for cell survival and for many cellular activities. Na,K-ATPase is highly expressed in glioblastoma cells (Mijatovic et al., 2008). Decreased expression of Na,K-ATPase β_1_-subunit has also been reported in various cancers (Rajasekaran et al., 1999, 2001; Espineda et al., 2003, 2004; Seligson et al., 2008; Rajasekaran and Rajasekaran, 2009). The effect of the Na,K-ATPase has also been studied in glioblastoma cells. Treatment with digoxin and ouabain, both specific Na,K-ATPase inhibitors, resulted in impaired proliferation in glioblastoma cell lines and the same cells exhibited an apoptotic phenotype (Joshi et al., 2011). Along with the identification of its differential expression in cancer cells and its important roles in cell survival, proliferation, adhesion and migration, the clinical potential of Na,K-ATPase modulators such as cardiac glycosides in oncology has drawn increasing interest in recent years (Newman et al., 2008; Prassas and Diamandis, 2008; Menger et al., 2012; Alevizopoulos et al., 2014; Wolle et al., 2014).

### Ion Carriers, Ion Channels and Ion Pumps in Cancer Cell Migration

Ion channels and pumps are the targets of many intracellular signaling pathways and can function as signal transducers themselves. Nearly every type of voltage-gated K^+^, Ca^2+^, and Na^+^ channel is regulated to some extent by phosphorylation of serine, threonine, or tyrosine residues on intracellular domains of the channel (Ismailov and Benos, 1995). Phosphorylation can alter channel gating properties, including voltage sensitivity and calcium sensitivity, thereby potentially altering the electrophysiological properties of a cell. While initially only studied in excitable cells, ion channels are now known to be important in supporting the basic biology of malignant and non-malignant cells including cell proliferation and tumor cell migration. The ability of cancer cells to migrate allows them to change position within the body and invade other tissues. As a primary tumor grows, it must undergo angiogenesis in order to support its metabolic needs. New blood vessels can also provide a route by which tumor cells can migrate through the body’s circulatory system, into a distant organ or tissue (Chambers et al., 2002). Tumor cells use migration mechanisms that are similar, if not identical to those in normal cells during normal physiological processes such as embryogenesis, wound healing, and neuronal migration. Migration requires cells to modify their shape and stiffness to interact with surrounding tissue, and results from a continuous cycle of interdependent steps. First, a moving cell becomes polarized, and, through Rac activation, a protrusion of the lamellipodium is formed at the cell’s leading edge, which attaches to the extracellular matrix (ECM). Next, regions of the cell body contract, generating a force that leads to movement of the cell (Schwartz and Horwitz, 2006). Invasive cancer cells develop similar invadopodia to explore and invade the surrounding ECM. Both the outgrowth of lamellipodia or invadopodia and the retraction of the rear end of the cell require local swelling and shrinkage, respectively. These changes in cell volume are regulated by local ion transport through the NHE1, the Na^+^, ${\text{HCO}}_{\text{3}}^{\text{−}}$ contransporter, the Na^+^, K^+^,2Cl^−^ cotransporter, chloride channels at the leading edge and tip of the outgrowing lamellipodium/invadopodium (Schwab et al., 1999; Stroka et al., 2014; Turner and Sontheimer, 2014). In breast cancer cells, repetitive cycles of local, NHE1-dependent swelling at the cell front and shrinkage at the rear end enable cells to move independently of actin polymerization and integrin-mediated adhesion. This may be the mechanism underlying the rounded tumor cell migration that allows cell movement through dense ECM without matrix degradation (Friedl and Alexander, 2011).

### Potassium Channels

The best characterized functions of potassium channels in facilitating cell migration are related to their ability to cause volume changes by affecting potassium flow (see (Turner and Sontheimer, 2014) for a comprehensive review). An early study linking potassium channels to cell migration was on transformed Madin-Darby canine kidney (MDCK-F) renal epithelial cell migration. The authors showed that specific application of the IK channel blocker charybdotoxin to the trailing edge, but not the leading edge, inhibits migration and increases overall cell volume (Schwab et al., 1999). Their results revealed that inhibition and activation of the IK channel elicited nearly identical effects on migration, F-actin content and intracellular calcium concentration as anisosmotic cell swelling or shrinkage. Since IK channel inhibition or activation elicits similar volume changes as anisotonicity, the authors suggested that cell volume is a link between K^+^ channel activity, the actin cytoskeleton and migration (Schwab et al., 1999). More recent studies showed that genetic suppression or pharmacological inhibition of BK channels impairs glioma cell migration. Specifically, Weaver et al. (2006) found that inhibition of BK channels reduced glioma cell migration across a transwell filter, suggesting that K^+^ efflux is essential for glioma cell migration. Interestingly, chronic application of a BK channel opener also resulted in impaired glioma cell migration, demonstrating the importance of controlling potassium channel activity for proper cell movement (Kraft et al., 2003). Another important potassium channel involved with cell migration is KCa3.1 (Sciaccaluga et al., 2010; Catacuzzeno et al., 2011, 2012), an IK channel involved in retraction of the cell’s trailing edge for forward movement (Schwab et al., 2012). Inhibition of KCa3.1 disrupts glioma cell chemotaxis towards multiple ligands (Cuddapah et al., 2013). Moreover Clotrimazole, a KCa3.1 channel blocker inhibits the growth of glioblastoma cells by arresting cells at the G1-S transition, and delays development of intracranial glioblastoma tumor formation (Liu et al., 2010). The SK channel was shown to control constitutive Ca^2+^ entry and cancer cell migration through interaction with the Ca^2+^ channel Ora1 in lipid rafts. Localization of the SK-Ora1 complex was essential to control cancer cell migration, and co-localization of the two channels was found in primary human tumors and bone metastases (Chantôme et al., 2013). Potassium channels can also regulate cancer cell migration via non-canonical functions. For example, the K_V_11.1 (HERG) channel can form a macromolecular complex with vascular endothelial growth factor receptor (VEGFR)-1 and β_1_-integrin, and assembly of this complex confers a pro-migratory phenotype in leukemia (Pillozzi et al., 2002). Colocalization of α_9_β_1_ integrin with the Kir4.2 channel in focal adhesions at the leading edge of migrating cells can promote local Kir4.2 channel activity to induce formation of a lamellipodium extension for migration. Moreover, the Kir channel inhibitor barium, or knockdown of the subunit Kir4.2, specifically inhibited α_9_-dependent cell migration (deHart et al., 2008; Schwab and Stock, 2014).

### Sodium Channels

Expression of VGSCs has been associated with cell motility and metastatic behavior. Grimes et al. (1995) found that the strongly metastatic Mat-LyLu cell line expressed VGSCs, while the related but weakly metastatic AT-2 cell line did not. Interestingly, incubation of the Mat-Ly-Lu cell line with tetrodotoxin, a specific VGSC blocker, reduced the invasive capacity of the cells *in vitro*. A similar pattern was found in analogous human prostate cancer cell lines PC-3 and human prostate adenocarcinoma (LNCaP), respectively (Laniado et al., 1997). Both Na_V_1.6 and Na_V_1.7 were significantly upregulated in prostate cancer cell lines (Shan et al., 2014). *In vitro* experiments have also shown that tetrodotoxin inhibits cancer invasion, proliferation and migration in PC-3 prostate cancer cells (Shan et al., 2014). More specifically, the effects of VGSC α- and β-subunit expression individually have been researched in various cancers. Expression of the VGSC α-subunit has been reported in several cancers including breast cancer, prostate cancer, neuroblastoma, and glioma (Brackenbury, 2012). In some of these malignancies, α-subunit protein and mRNA expression correlated with metastatic potential. For example, VGSC was found to increase in line with metastatic potential in the LNCaP progression model of prostate cancer cells (Bennett et al., 2004). Conversely, in gliomas, the mRNA level of VGSC α-subunits is inversely correlated with malignancy grade (Schrey et al., 2002). The role of VGSC β-subunits has also been investigated. The β_1_-subunit is highly expressed in weakly metastatic breast cancer (MCF-7) cells, and it enhances adhesion while inhibiting migration in a transwell assay (Chioni et al., 2009). On the other hand, much lower levels of β_1_ were found in strongly metastatic breast cancer MDA-MB-231 cells. Overexpression of β_1_ in these cells increased cell-cell adhesion, induced process outgrowth, and reduced migration in wound healing assays (Chioni et al., 2009). Overexpression of β_2_ in LNCaP cells increased adhesion, process outgrowth, migration, and invasion (Jansson et al., 2012). Thus, it seems that β_1_ and β_2_ play slightly different functional roles in different cancer cells. It is important to note that β-subunits play a critical role in regulating adhesion and migration in excitable cells like neurons, where they are normally expressed. For example, β_1_ enhances neurite outgrowth and neuronal path finding during early postnatal development of the central nervous system (Davis et al., 2004). β_1_-mediated neurite outgrowth in cerebellar granule cells requires fyn kinase, the cell adhesion molecule contactin, and is dependent on Na_V_1.6 activity (Brackenbury et al., 2008).

### Chloride Channels

Chloride channels are critical in maintaining cell volume, and therefore play an important role in regulating migration of cancer cells. Migratory glioma cells are elongated, suggesting that invading glioma cells undergo cell volume changes to move through the extracellular space (Sontheimer, 2004). Blocking Cl^−^ channels inhibits changes in cell volume and inhibits cell migration (Watkins and Sontheimer, 2011). Inhibition of NKCC1, which maintains an outward gradient for Cl^−^, also impairs motility of glioma cells *in vitro* (Haas and Sontheimer, 2010). Non-specific Cl^−^ channel inhibitors decrease cell migration *in vitro* (Soroceanu et al., 1999). However more specifically, shRNA mediated knockdown of ClC-3 decreased *in vitro* glioma cell migration (Cuddapah and Sontheimer, 2010) and Chlorotoxin, which reduces membrane expression of ClC-3, inhibited migration *in vitro* and *in vivo* (Sontheimer, 2008).

#### Calcium Channels

Changes in intracellular Ca^2+^ concentrations play an important role in cancer cell migration and tumor metastasis, and multiple Ca^2+^ channels have been linked to cancer cell migration. Inhibition of the TRPM7 channel resulted in reversal of epidermal growth factor (EGF)-induced migration of a lung cancer cell line and inhibited basal migration of the cells in the absence of EGF (Gao et al., 2011). High levels of TRPM7 expression predicted poor outcome in breast cancer patients and was functionally required for metastasis in a mouse xenograft model of human breast cancer (Middelbeek et al., 2012). Inhibition of TRPC6 activity showed reduced glioma cell growth, G2 phase cell cycle arrest, and reduced growth of subcutaneously and intracranially implanted gliomas, as well as increased survival of mice *in vivo* (Ding et al., 2010). Another study involving TRPC6 revealed that TRPC6 knockdown inhibited glioma cell invasion and angiogenesis, and they concluded that TRPC6 is required for development of the aggressive glioma phenotype (Chigurupati et al., 2010). Like NHE1 or NCKK, TRPC1 channels were localized to lipid raft domains at the leading edge of migrating glioma cells, and shRNA-mediated TRPC1 knockdown resulted in loss of EGF-induced glioma cell migration (Bomben et al., 2011).

#### Ion Pumps

The Na,K-ATPase has been linked to cancer cell motility and migration *in vitro*. Repletion of Na,K-ATPase β_1_-subunit in Maloney Sarcoma virus transformed MDCK (MSV-MDCK) cells induced lamellipodia and suppressed motility in a phosphatidylinositol 3-kinase (PI3-kinase) dependent manner, and protein kinase C was involved upstream of PI3-kinase in the suppression of β_1_-subunit mediated cell motility in carcinoma cells (Rajasekaran et al., 2001; Barwe et al., 2005, 2006). The Na,K-ATPase α_1_-subunit associated with the regulatory subunit of PI3-kinase and β_1_-subunit bound to anexin II, and these molecular interactions localized activated PI3-kinase at lamellipodia and suppressed cell motility in MSV-MDCK cells, independent of Na,K-ATPase ion transport activity (Barwe et al., 2005). Changes in Na,K-ATPase subunit expression were also found in various other cancers (Rajasekaran et al., 1999; Espineda et al., 2003; Lefranc et al., 2008; Mijatovic et al., 2008; Seligson et al., 2008; Rajasekaran and Rajasekaran, 2009). Ouabain, an Na,K-ATPase inhibitor, inhibited EGF induced medulloblastoma cell motility in a wound healing assay, prevented formation of EGF-induced actin stress fibers, and inhibited EGF-induced phospho-focal adhesion kinase (FAK) localization to the lamellipodia of medulloblastoma cells (Wolle et al., 2014). The Na,K-ATPase β_2_-subunit has also been linked to cancer cell motility, specifically in glioblastoma multiforme. Na,K-ATPase β_2_-subunit expression inhibits invasion of glioblastoma multiforme cells, and its downregulation increases invasion in glial cells (Sun et al., 2013). Another group found that inhibition or down regulation of Na,K-ATPase induced a cell death with characteristics of both apoptosis and necrosis, and disruption of K^+^ homeostasis was important in induction of apoptosis in human glioblastoma cells (Chen et al., 2014).

#### Ion Channels and Cell Signaling

There is increasing evidence that ion channels can form signaling complexes with cell adhesion proteins including integrins. Integrins are a family of membrane-spanning glycoproteins that link ECM to the cytoskeleton. They are composed of α-β heterodimers with extracellular domains that bind ECM proteins and short cytoplasmic tails that associate with focal adhesion proteins. Integrins form signaling complexes at cell adhesion sites with other membrane receptors. Adaptor proteins such as Cas, Crk, and paxillin contribute to formation of adhesive complexes, which comprise focal adhesions. Downstream signaling pathways are then triggered by several enzymes including focal adhesion kinasse (FAK), the tyrosine kinase Src, and GTPases such as Ras and Rho (Brown, 2002). Evidence suggests that cellular processes that require activation of adhesion proteins such as cell differentiation, neurite extension and migration depend on ion channel activity. Multiple studies have demonstrated the crosstalk between ion channels and cell adhesion molecules. One of the first studies to reveal the formation of macromolecular complexes between integrins and ion channels came when McPhee et al. (1998) found that the G protein-gated inwardly rectifying K^+^ channels (GIRKs) contain an Arginine-Glycine-Aspartate (RGD) sequence on their extracellular loop. RGD sequences mediate interaction between ECM proteins and integrins and are absent on the extracellular side of other K^+^ channels. Further evidence showed that β_1_ integrin associates with the Na,K-ATPase and voltage gated Ca^2+^ channels (Shakibaei and Mobasheri, 2003). In PC12 cells, the shift to a neuronal phenotype induced by neural cell adhesion molecules and N-cadherins is mediated by Ca^2+^ channels (Doherty et al., 1991). Integrins were observed to regulate neurite extension in neuroblastoma cells by activating K^+^ channels (Arcangeli et al., 1993).

Ion pumps can also activate cellular signaling pathways. For example, in addition to its ion pumping function, Na,K-ATPase can also act as a signal transducer. When cells are exposed to drugs known to be specific inhibitors of Na,K-ATPase (such as ouabain, digoxin, and digitoxin), multiple intracellular signaling pathways are activated, including the Epidermal growth factor receptor (EGFR)/Src-Ras-Erk pathway and the PI3K-PDK-Akt pathway (Pierre and Xie, 2006; Reinhard et al., 2013). Ouabain can activate the Ras and Erk signaling pathways. Src is necessary for ouabain-induced activation of the EGFR/Ras-Erk pathway, and activation of PI3-kinase and Akt by ouabain leads to fibroblast cell proliferation (Wu et al., 2013). As mentioned earlier, the Na,K-ATPase β_1_-subunit suppresses cell motility by cross talk between the two subunits of Na,K-ATPase with the PI3-kinase signaling pathway (Barwe et al., 2005). However, in medulloblastoma cells, ouabain did not transactivate EGFR as has been reported in other cell lines, and ouabain inhibited EGF-induced Erk1/2 and Akt activation (Wolle et al., 2014).

#### Ion Channels, Proliferation and the Cell Cycle

During development, tissues grow by proliferation, with some cell types able to divide throughout life. Cell proliferation is highly regulated in order to assure proper coordination of cell number. Cancer cells divide and proliferate faster than normal cells, resulting in tumor development. A role for ion channels in cell proliferation has been found in multiple cell types and cancers. Studies have found that some ion channel blockers also inhibit cell cycle progression and arrest cells at distinct stages of the cell cycle (Becchetti, 2011). For example, different potassium channels show cell-cycle-dependent variations in expression and activity (Pardo et al., 1998). Potassium channel blockers and depolarizing agents inhibit cell proliferation in oligodendrocyte progenitors in cell culture and cerebellar tissue slices by inducing G1 arrest through accumulation of p27 and p21, two cyclin dependent kinase (CDK) inhibitors involved in cell proliferation (Ghiani et al., 1999). Inhibition of the voltage gated K^+^ channels K_V_1.3 and K_V_1.5 decreased proliferation of glioma cells (Pardo, 2004). For the K_V_1.3 channel, this affect on cell proliferation was due to interaction with the signaling adaptor protein 14-3-3 (Rajan et al., 2002). Also, inhibition of ATP-sensitive K^+^ channels (K_ATP_) decreased proliferation *in vitro* and slowed tumor formation in xenografts, with cells arrested at G0/G1 phase of the cell cycle (Huang et al., 2009). Knockdown of Kir2.2 induced a significant increase in reactive oxygen species accompanied by cell cycle arrest. Moreover, pre-established tumors reduced in size after injection of siRNA targeting Kir2.2 (Lee et al., 2010). EAG channels are involved in cell cycle control, and inhibition of EAG expression in multiple cancer cell lines leads to reduction in cell proliferation (Pardo et al., 1999). Kir4.1 is also an important channel in regulating cell division (Turner and Sontheimer, 2014). Blocking the Kir4.1 channel in astrocytes induced proliferation, while overexpression in glioma cells arrested proliferation (Higashimori and Sontheimer, 2007). A role of BK channels in glioblastoma proliferation has been suggested (Chin et al., 1997; Wang, 2004). BK channel inhibition arrests glioma cells in S phase of the cell cycle, and causes a dose and time dependent decrease in cell number as early as 72 h after exposure (Weaver et al., 2004). Nevertheless, other groups suggested that some of the inhibitors of BK channels may regulate cell proliferation through off-target mechanisms (Abdullaev et al., 2010).

Calcium signaling also plays an important role in regulation of the cell cycle. Cells progressing from G1 to S require both extracellular calcium and functional membrane calcium channels in order to activate downstream signaling (Taylor et al., 2008). Partial inhibition of plasma membrane Ca^2+^ ATPase (PMCA) mediated calcium efflux reduced proliferation of MCF-7 breast cancer cells. An antisense construct directed towards PMCA lead to changes in cell cycle control kinetics, with a prolonged G2 phase that occurs with reduced Ca^2+^ efflux (Lee et al., 2005). Knockdown of TRPC6 expression inhibits glioma growth, invasion, and angiogenesis, and is an important mediator of glioblastoma tumor growth *in vitro* and *in vivo* (Chigurupati et al., 2010).

In addition, changes in sodium channels impact tumor cell proliferation. For example, the nNa_V_1.5 channel, a neonatal form of VGSC, was upregulated in human brain astrocytoma and positively correlated with the degree of malignancy while reduced nNa_V_1.5 expression significantly suppressed the proliferation of astrocytoma cells (Xing et al., 2014). Thus, modulation of various ion channels has profound effects on regulating cell cycle control and proliferation of cancer cells.

#### Therapeutic Potential

Most cancers are treated by surgical resection, chemotherapy, and radiation therapy. However due to the prevalence of cancer relapse with treatment resistance, novel molecular targets must be identified for the development of alternative therapies. Despite the growing evidence of aberrant expression and function of ion channels in oncology, development of cancer treatment using ion channel targeting compounds is still at an early stage. Studies have shown that some VGSC blockers can inhibit cell behaviors associated with metastasis. For example, the anticonvulsant phenytoin suppressed migration of prostate cancer cells (Fraser et al., 2003), and also inhibited migration and invasion in metastatic breast cancer cells (Yang et al., 2012). Riluzole, which is both a VGSC blocker and metabotropic glutamate receptor inhibitor, reduced the breast cancer tumor volume in mice, and suppressed metabolic activity of tumors in patients with stage III and IV melanoma (Yip et al., 2009). The K_V_10.1 EAG1 channel has been closely studied as a target for cancer therapy, since EAG1 overexpression is associated with tumorigenic potential, and its expression is correlated with poor patient survival in multiple cancer types (Pardo and Stühmer, 2008). The small molecule EAG1 blocker astemizole reduced EAG1-expressing cancer cell growth *in vitro* and *in vivo* (Downie et al., 2008). A highly specific extracellular epitope-targeting EAG1 monoclonal antibody exhibited antitumor activity in a mouse model (Gómez-Varela et al., 2007). T-type Ca^2+^ channels have also been suggested as a potential therapeutic target for certain brain tumors. Mibefradil, a calcium channel inhibitor, was found to inhibit proliferation in human glioma and neuroblastoma cells, and overexpression of T-type Ca^2+^ channel protein doubled the proliferation rate while antisense treatment reduced the proliferation rate of these cells (Panner et al., 2005). The Na,K-ATPase is another potential target for cancer therapy. Human glioblastoma cells that are resistant to the chemotherapy drug temozolomide (TMZ) show a higher expression of Na,K-ATPase α_2_- and α_3_-subunits compared to TMZ-sensitive cells and astrocytes. These cells were also sensitive to ouabain at low concentrations, and knockdown of the α_3_-subunit sensitized the cells to TMZ (Chen et al., 2014). Judging from the large number of ion channels that are altered in cancers, it is clear that understanding more about ion channels and pumps and how they contribute to cancer progression could reveal new therapeutic targets for many different cancers. The findings discussed above provide proof of principle that ion channels are effective targets for cancer therapy.

## Conclusions

Increasing evidence suggests that ion channels and pumps are involved in the regulation of cell proliferation and migration, and channel proteins have been shown to form macromolecular complexes with cell adhesion molecules and other signaling proteins. As these roles of ion channels and pumps are further elucidated, it is being increasingly suggested that regulation of ion channels and pumps could contribute to cancer progression. Here we discussed that aberrant expression and function of several types of ion channels and pumps have been found in multiple cancers including brain cancer, and in particular, glioblastoma. Knowing that these proteins are involved in multiple malignant characteristics of multiple cancers, ion channels and pumps could be potential targets for therapy.

## Conflict of Interest Statement

The authors declare that the research was conducted in the absence of any commercial or financial relationships that could be construed as a potential conflict of interest.

## Abbreviations

ASIC, Acid-sensing ion channel; BK, Big conductance channel; CDK, Cyclin dependent kinase; CFTR, Cystic fibrosis transmembrane conductance regulator; EAG1, Voltage gated eag related subfamily H, member 1; ECM, Extracellular matrix; EGF, Epidermal growth factor; EGFR, Epidermal growth factor receptor; ER, Endoplasmic reticulum; FAK, Focal adhesion kinase; GIRK, G protein-gated inwardly rectifying K^+^ channel; HER2, Human epidermal growth factor receptor 2; IK, Intermediate conductance channel; K2P, Two-pore domain potassium channel; K_ATP_, ATP-sensitive K^+^ channels; KCa, Calcium-activated potassium channel; Kir, Inward rectifying potassium channel; KV, Voltage-gated potassium channel; MDCK-F, transformed Madin-Darby canine kidney; Na, K-ATPase, Sodium potassium ATPase; NHE1, Na^+^/H^+^ exchanger; NKCC, Na^+^, K^+^, 2Cl^−^ cotransporter; PI3-kinase, Phosphatidylinositol 3-kinase; PMCA, Plasma Membrane Ca^2+^ ATPase; RGD, Arginine-Glycine-Aspartate; SERCA, Sarcoplasmic and endoplasmic reticulum Ca-ATPase; SK, Small conductance channel; TMZ, Temozolomide; TRP, Transient receptor potential channel; VEGFR, Vascular endothelial growth factor receptor; VGSC, Voltage gated sodium channel.
